# The Microbiology Bench Companion

**DOI:** 10.3201/eid1402.071349

**Published:** 2008-02

**Authors:** Cathy A. Petti

**Affiliations:** * University of Utah School of Medicine, Salt Lake City, Utah, USA

Diagnostic testing for infectious diseases is an increasingly complex area of laboratory medicine. The microbial community continues to evolve and adapt to changing environmental influences, and the distribution of human pathogens has become more global. Our recognition of the spectrum of microorganisms that cause invasive human disease has exploded with the use of culture-independent methods to detect and characterize pathogens. Clinicians, epidemiologists, and public health officials can benefit from consultative interactions with laboratory professionals to assist with optimizing diagnostic test options and interpretation of test results. For technologists without access to board-certified laboratory professionals who can guide the work-up of infectious agents in a microbiology laboratory, a concise guide can be extremely beneficial.

In this handbook, J. Michael Miller, a highly experienced clinical microbiologist, distills a great deal of information into 120 pages, largely formatted as tables and flowcharts. Molecular methods for detecting or identifying microorganisms are notably absent, which may reflect the author’s intent to address readers who perform conventional diagnostic tests only. The handbook is divided into 3 sections. Section 1 focuses on routine laboratory bench algorithms for identifying bacteria, fungi, and parasites. Although most of this information can be found in clinical microbiology textbooks, the flowcharts are an easy reference, especially for medical technologists. Section 2 is entirely formatted with tables listing clinical syndromes (e.g., cellulitis, pneumonia, gastroenteritis) and their possible infectious causes. The lists of etiologic agents are extensive, yet practical, and would be of most benefit to infection control practitioners, nurses, and laboratory technologists. Section 3 is devoted to therapeutic choices for various microorganisms and is intended to inform laboratorians of treatment options; it is not a guide for therapeutic decision-making by clinicians. Although certain taxonomic classifications and susceptibility guidelines do not reflect the current standard, most information, such as annotated remarks for pathogens classified as select agents, is timely.

The author valiantly furnishes us with pearls and nuances of clinical microbiology in this clear and concisely written handbook. The task was Herculean. As the author aptly notes in the introduction, no handbook can capture every parameter or indication for identification of all clinically relevant microorganisms and describe these microorganisms in public health or patient-centered contexts. Overall, the author delivers a wealth of information that can benefit technologists and healthcare practitioners in regions with limited access to professionals who specialize in clinical microbiology or infectious diseases.

